# Human Beta-Defensin-1 Suppresses Tumor Migration and Invasion and Is an Independent Predictor for Survival of Oral Squamous Cell Carcinoma Patients

**DOI:** 10.1371/journal.pone.0091867

**Published:** 2014-03-21

**Authors:** Qi Han, Ruinan Wang, Chongkui Sun, Xin Jin, Dongjuan Liu, Xin Zhao, Lili Wang, Ning Ji, Jing Li, Yu Zhou, Ling Ye, Xinhua Liang, Lu Jiang, Ga Liao, Hongxia Dan, Xin Zeng, Qianming Chen

**Affiliations:** State Key Laboratory of Oral Diseases, West China Hospital of Stomatology, Sichuan University, Chengdu, China; Karolinska Institutet, Sweden

## Abstract

**Background:**

Human beta-defensin-1 (hBD-1) has recently been considered as a candidate tumor suppressor in renal and prostate cancer. The aim of this study was to investigate the role of hBD-1 in the progression of oral squamous cell carcinoma (OSCC) and its potential as diagnostic/prognostic biomarker and therapeutic target for OSCC.

**Methods:**

HBD-1 expression in tissues at different stages of oral carcinogenesis, as well as OSCC cell lines was examined. HBD-1 was overexpressed in HSC-3, UM1, SCC-9 and SCC-25 cells and subjected to cell growth, apoptosis, migration and invasion assays. Tissue microarray constructed with tissues from 175 patients was used to examine clinicopathological significance of hBD-1 expression in OSCC.

**Results:**

HBD-1 expression decreased from oral precancerous lesions to OSCC and was lower in OSCC with lymph node metastasis than those without metastasis. *In vitro*, the expression of hBD-1 was related to the invasive potential of OSCC cell lines. Induction of exogenous expression of hBD-1 inhibited migration and invasion of OSCC cells, probably by regulation of RhoA, RhoC and MMP-2; but had no significant effect on proliferation or apoptosis. In a cohort of patients with primary OSCC, cases with no expression of hBD-1 had more chance to be involved in lymph node metastasis. Eventually, the positive expression of hBD-1 was associated with longer survival of patients with OSCC, and multivariate analysis and ROC curve analysis confirmed hBD-1 positivity to be an independent prognostic factor of OSCC, especially OSCC at early stage.

**Conclusions:**

Overall, these data indicated that hBD-1 suppressed tumor migration and invasion of OSCC and was likely to be a prognostic biomarker and a potential target for treatment of OSCC.

## Introduction

Oral squamous cell carcinoma (OSCC) is one of the most common oral and maxillofacial malignancies, causing around 130,000 deaths each year worldwide [1]. Despite the advances in early detection of oral precancerous lesions and treatment of OSCC in recent years[2]–[5], the overall survival rate of OSCC is still not promising, probably due to the lack of understanding of the molecular mechanisms of carcinogenesis. Therefore, it is necessary to search for novel targets for treatment of this disease and to identify molecular markers associated with the progression of this disease.

Human beta-defensin-1 (hBD-1) is a small cationic peptide encoded by human *DEFB1* gene located on the short arm of the chromosome 8 [6]. It is mainly expressed in epithelial tissues [7]–[12]. Recently, it was reported that other than its antimicrobial and chemotactic activities [13], [14], hBD-1 also showed characteristics as a potential tumor-suppressor. Gene mutation of *DEFB1* or cancer-specific loss of hBD-1 expression has been reported in basal cell carcinoma, renal cell carcinoma and prostate cancer [15]–[17]. Transduction of *DEFB1* gene or treatment with recombinant hBD-1 led to inhibition of cell growth and apoptosis of tumor cells [17], [18]. Therefore, the researchers proposed that hBD-1 might be a potential tumor suppressor. Similarly, *DEFB1* gene expression was observed in normal oral mucosa [7], [19]–[21], while in some precancerous lesions, OSCC tissues and cell lines, the expression was decreased [21]–[23]. *In vitro* studies showed that recombinant hBD-1 had inhibitory effect on the growth of some OSCC cell lines [24]. Taken together, these studies indicate that hBD-1 may also have some suppressive effect on oral carcinogenesis, but the effect of hBD-1 on other behaviors of OSCC cells and the clinicopathological significance of hBD-1 in OSCC is still unclear.

In the current study, we investigated the expression of hBD-1 protein in oral epithelia at different stages of oral carcinogenesis, the impact of hBD-1 on migration, invasion, proliferation and apoptosis of OSCC cells, as well as the clinicopathological significance of hBD-1 in a large group of patients with primary OSCC. We have shown that hBD-1 suppresses tumor migration and invasion of OSCC and is likely to be a prognostic biomarker and a potential target for treatment of OSCC.

## Materials and Methods

### Clinical tissue samples

This study was carried out under the approval and supervision of the Ethnic Committee of Sichuan University and written informed consent was obtained from each patient. Sixty-two formalin-fixed paraffin-embedded tissue blocks was collected in order to detect hBD-1 expression in normal oral mucosa, precancerous lesion oral leukoplakia (OLK) and primary OSCC. Among them, 30 OSCC samples and 17 OLK samples were collected during surgery or biopsy, 15 normal oral mucosa samples were obtained from extra tissue from orthognathic or implant surgery. Primary OSCC samples used for hBD-1 staining on tissue microarrays were obtained from 175 patients who underwent curative surgery between 2002 and 2009 in West China Hospital of Stomatology, Sichuan University (Chengdu, China), with a mean follow-up time of 35 months (median, 33 months; range 2∼102 months). All tumors were staged according to the TNM classification system of the International Union against Cancer [25]. The survival time of each patient was calculated from the day of surgery until the time of cancer-related death or end of the follow-up, while death due to other reasons was considered as censored data.

### Immunohistochemistry

A mouse monoclonal antibody against hBD-1 (Abcam) was used for immunohistochemistry. The specificity of the antibody was tested using dot blot (Figure S1). Sections were rehydrated and antigen retrieved with Tris-EDTA buffer (pH 9.0). Slides were peroxidase blocked with 3% hydrogen peroxide solution for 10 minutes and then blocked using 5% bovine serum albumin (Sigma) for 30 minutes. Slides were subsequently incubated with primary antibody against hBD-1 (Mouse monoclonal, Abcam, 1∶200) or IgG negative control (Mouse IgG, Abcam, 1∶20) for 30 minutes and detected with ChemMate DAKO EnVision Detection Kit (DAKO). Slides were counterstained with haematoxylin, dehydrated and mounted. Human skin and salivary gland samples were used as positive control for hBD-1 [8], [11].

The staining was scored by three independent investigators without any knowledge of the clinicalpathological data. The following criteria was used to score the staining: negative, no detectable staining; weak, brown staining in less than 50% of tumor cells; strong, brown staining in more than 50% of tumor cells. The staining intensity was acceptable if two or more investigators independently defined it as such.

### Cell lines and cell culture

Four human OSCC cell lines, HSC-3, UM1, SCC-9 and SCC-25 were used in this study. HSC-3 (JCRB0623) was purchased from the cell bank of Japanese Collection of Research Bioresource (JCRB, Shinjuku, Japan). UM1 [26] was provided by Dr. Xiaofeng Zhou (Center for Molecular Biology of Oral Diseases, College of Dentistry, University of Illinois at Chicago). SCC-9 (CRL-1629) and SCC-25 (CRL-1628) were purchased from American Type Culture Collection (ATCC, Manassas, USA). Human immortalized oral keratinocyte cell line HOK16E6E7 was provided by Dr. Xuan Liu (Charles R. Drew University of Medicine and Science) [27]. All cells were cultured in appropriate media with 10% fetal bovine serum (PAA Laboratories) in a humidified incubator at 37°C with 5% CO_2_.

### Semi-quantitative real-time PCR

Total RNA was extracted from cultured cells using Trizol reagent (Invitrogen) and reversed transcribed using PrimeScript RT reagent kit (Takara). Semi-quantitative real-time PCR was done using the SYBR Premix Ex Taq II kit (Takara) on a 7300 real-time PCR system (Applied Biosystems) with *DEFB1* gene-specific primers (5′-CCTTCTGCTGTTTACTCTCTGC-3′ and 5′-GAATAGAGACATTGCCCTCC- AC-3′) or with glyceraldehyde- 3-phosphate dehydrogenase (GAPDH) specific primers (5′-CTTTGGTATCGTGGAAGGACTC-3′ and 5′-GTAGAGGCAGGGATG- ATGTTCT-3′) as an internal control.

### Enzyme-linked immunosorbent assay

HBD-1 expression in cell culture supernatant was determined in triplicates by enzyme-linked immunosorbent assay (ELISA) using Human BD-1 ELISA Development Kit (Peprotech) according to the manufacturer's protocol. Data was read with a spectrophotometer (Thermo) at 450 nm.

### Migration and Invasion assay

5×10^4^ cells of different groups were re-suspended with serum-free Dulbecco's modified eagle medium (DMEM, Gibco, Invitrogen) and then seeded in a cell culture insert (pore size 8 µm, BD Biosciences) for 24-well plate in triplicates. DMEM containing 10% fetal bovine serum was added to the lower chamber. 24 hours later, cells in the upper chamber were wiped using a cotton stick and cells migrated to the other side of the chamber were fixed with 70% methanol and stained by haematoxylin.

Procedures of invasion assay were similar to migration assay except for that BioCoat Matrigel Invasion Chambers (BD Biosciences) were used instead of uncoated inserts, and cell number and incubation time were doubled.

### Plasmids and lentiviral infection

HSC-3, UM1, SCC-9 and SCC-25 cells with stable expression of hBD-1 were generated using a lentivirus-delivered eukaryote expression plasmid. Human *DEFB1* (NM_005218) cDNA was cloned to the pGC-FU-3FLAG-IRES-Puromycin Vector, then lentivirus delivering the *DEFB1* gene (pGCHBD-1) or empty vector (pGCcontrol) was prepared (provided by Genechem Corporation Ltd., Shanghai, China). HSC-3 and UM1 cells were infected with either pGCHBD-1 or pGCcontrol, after selection in puromycin, expression of hBD-1 was verified by real-time PCR and ELISA.

### Cell proliferation, colony formation and apoptosis assay

HSC-3, UM1, SCC-9 and SCC-25 cells stably transfected with pGCHBD-1 or pGCcontrol plasmids were seeded in 96-well plate at the concentration of 1×10^3^ cells/well. Every 24 hours after seeding, relative number of living cell was assessed using Cell Counting Kit-8 (CCK-8, Dojindo). Meanwhile, HSC-3, UM1, SCC-9 and SCC-25 cells were treated with 50 µg/ml recombinant hBD-1 (Peprotech), and cell viability with or without hBD-1 treatment was also assessed by CCK-8 assay.

For colony formation assay, cells were seeded in 6-well plate at the concentration of 3×10^2^ cells/well. 14 days after seeding, cells were fixed with 4% paraformldehyde and then stained with crystal violet. Colony formation rate = colony number/cells seeded×100%.

The effect of exogenous expression of hBD-1 on apoptosis of HSC-3, UM1, SCC-9 and SCC-25 cells was assessed by Annexin-V fluorescein isothiocyanate (FITC) and propidium iodide (PI) double staining. Briefly, 48 hours after transfection with pGCHBD-1 or pGCcontrol plasmids, 2×10^5^ trypsinezed cells from each group were collected and stained according to the instructions of the Annexin V-FITC Apoptosis Detection Kit (R&D Systems) and analyzed by a flow cytometer (Beckman Coulter). The data was presented as dot plots showing fluorescence intensity of Annexin-V FITC and PI. The percentage of apoptotic cells (Annexin-V+/PI− and Annexin-V+/PI+) was calculated.

### Western blot

Proteins from each group were extracted and applied to western blot analysis as described elsewhere [28], with primary antibodies specific for vimentin (mouse monoclonal, 1∶500, Abcam), RhoA (mouse monoclonal, 1∶250, Abcam), RhoC (rabbit polyclonal, 1∶500, Abcam) and MMP-2 (rabbit polycolonal, 1∶500, Abcam). GAPDH antibody (rabbit polycolonal, 1∶5,000, Trevigen) was used as an internal control.

### Tissue microarray

In order to verify the clinicopathological significance of hBD-1 expression in a large group of OSCC patients, tissue microarrays (TMA) were constructed with formalin-fixed, paraffin-embeded tissue blocks from 175 patients with primary OSCC. In brief, the area for tissue cores was selected under microscope with a corresponding section with Hematoxylin & Eosin staining. Tissue cores (diameter, 1.5 mm) were taken from each donor block and then placed into a receiver paraffin block using a special tissue arrayer. The TMA block was then cut into 4 µm sections and used for immunohistochemical staining of hBD-1 protein as described above.

### Statistical analysis

Statistical Package for Social Science (SPSS) version 17.0 for windows was used to analyze the data. Student's *t* test was used to compare the difference of migration, invasion, proliferation, colony formation of cells from different groups. Fisher's exact test was used to analyze the independence of hBD-1 expression and clinicopathological characteristics. The Kaplan-Meier survival analysis was applied to estimate cancer-specific survival rates, the difference between different groups were examined using log-rank test. Factors associated with the prognosis were analyzed using Cox proportional hazard regression model with a step-down mode. Receiver operating characteristic (ROC) curve analysis was used to determine the discrimination of hBD-1 expression as a predictor for 3-year survival of patients with OSCC. For all the statistical analysis, *P* value less than 0.05 was considered significant.

## Results

### HBD-1 expression decreased from oral precancerous lesions to OSCC and was lower in OSCC with lymph node metastasis than those without metastasis

**Figure 1:**
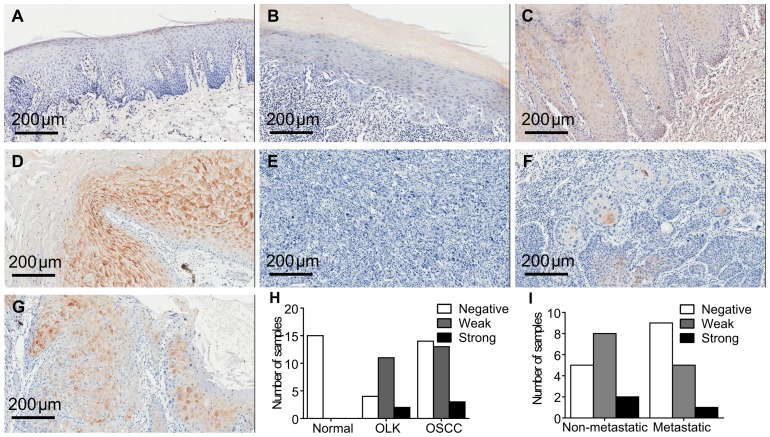
Immunostaining of hBD-1 protein in different tissue samples. A) Examples of typical images of hBD-1 in normal oral mucosa (negative); B) OLK (negative); C) OLK (weak); D) OLK (strong); E) OSCC (negative); F) (weak); G) OSCC (strong). H) Number of samples with negative, weak and strong staining of hBD-1 in normal oral mucosa, OLK and OSCC. I) Percentage of negative, weak and strong staining of hBD-1 in OSCC with or without metastasis.

To confirm the expression pattern of hBD-1 in different stages of oral carcinogenesis, we first examined the expression of hBD-1 protein in 15 normal oral mucosa, 17 OLK and 30 OSCC specimens by immunohistochemistry. None of the normal oral mucosa samples showed any detectable hBD-1 expression; while cytoplasmic staining of hBD-1 was detected in some OLK and OSCC samples (Figure 1A–G). There was a higher percentage of hBD-1 staining in OLK (13 in 17 samples) than in OSCC (16 in 30 samples) (P = 0.0.284; Figure 1 H). Furthermore, OSCC without metastasis also had slightly higher percentage of hBD-1 staining (10 in 15 samples) than OSCC with metastasis (6 in 15 samples) (P = 0.338; Figure 1 I).

### HBD-1 expression in OSCC cell lines was negatively correlated with the migration and invasion ability of OSCC cells

**Figure 2:**
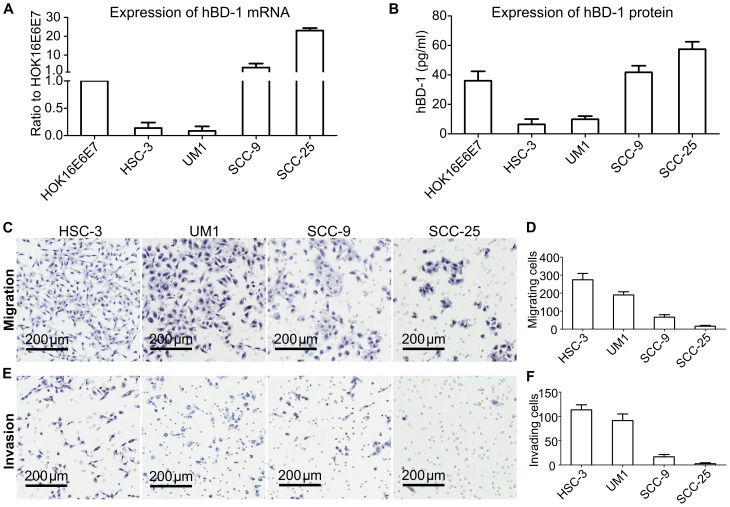
OSCC cell lines with low hBD-1 expression tended to have higher invasive potential. A) hBD-1 mRNA expression level in OSCC cell lines. B) hBD-1 protein expression level in the supernatant of OSCC cell lines. Immortalized oral keratinocyte HOK16E6E7 was used as control. C–D) Assays showing the relative migration ability of OSCC cell lines. Haematoxylin staining (C) and the relative numbers of cells migrated to the lower surface of the cell culture insert (D). E–F) Assays showing the relative invasion ability of OSCC cell lines. Haematoxylin staining (E) and the relative numbers of cells migrated to the lower surface of the matrigel-coated cell culture insert (F). All assays were carried out three times in triplicates.

As OSCC samples without metastasis tended to have higher expression of hBD-1, the relationship of hBD-1 with migration and invasion of OSCC cells was suspected. The expression of hBD-1 in four OSCC cell lines with different invasive potentials was examined (Figure 2 A, B), human immortalized oral keratinocyte HOK16E6E7 was used as control. Two OSCC cell lines, HSC-3 and UM1, showed low expression of hBD-1, while cell lines SCC-9 and SCC-25 had relatively higher expression of hBD-1. The migration and invasion potential of these four OSCC cell lines was then analyzed. The results showed that cell lines with higher expression of hBD-1 tended to have lower potential of migration (Figure 2 C, D) and invasion(Figure 2 E, F) in vitro.

### Exogenous expression of hBD-1 inhibited migration and invasion of OSCC cells but had no significant effect on the proliferation and apoptosis on OSCC cells in vitro

**Figure 3:**
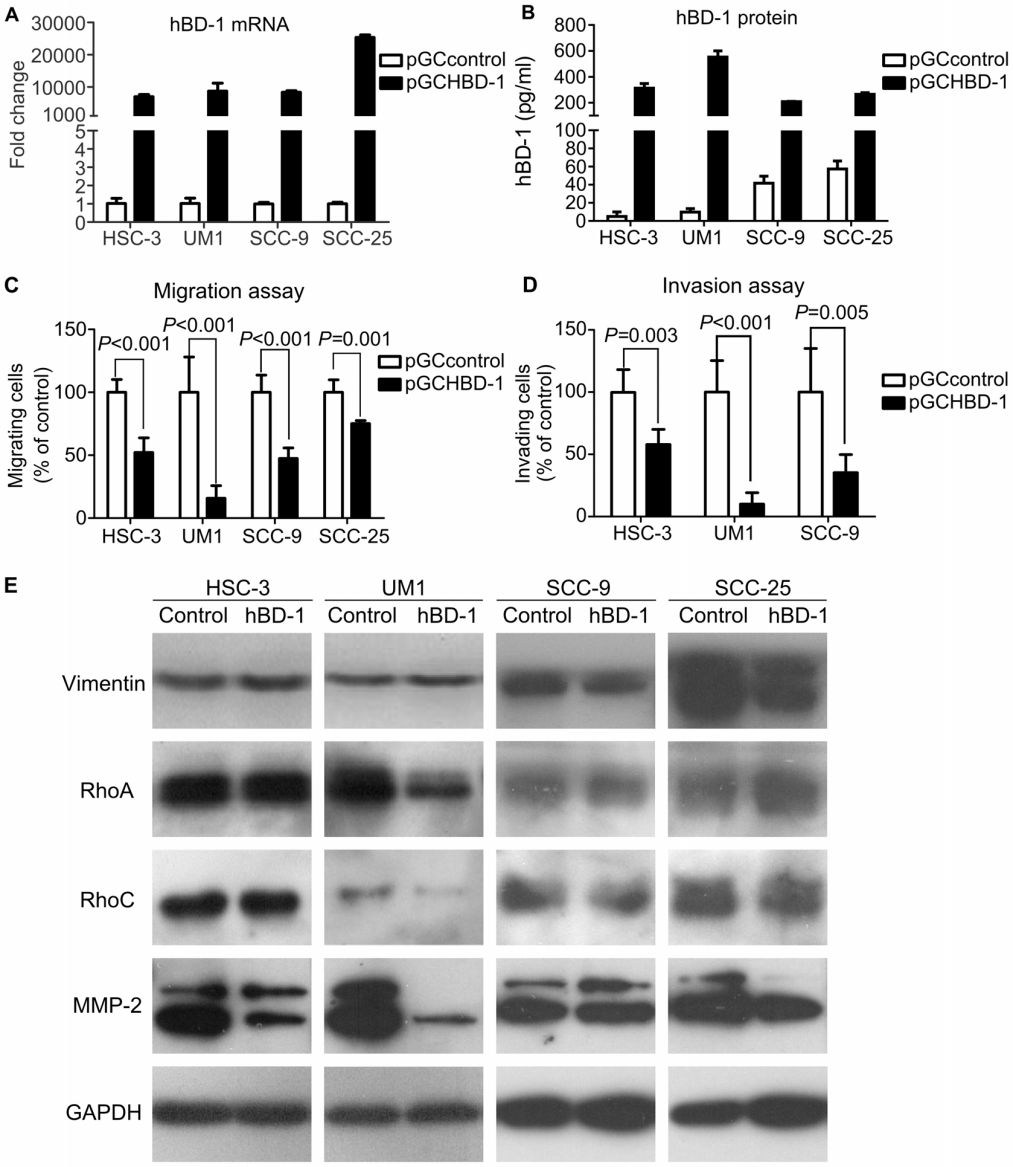
Effect of exogenous expression of hBD-1 on the migration and invasion of HSC-3, UM1, SCC-9 and SCC-25 cells. Expression of hBD-1 mRNA (A) and protein (B) was enhanced after transfection with lentivirus-delivered pGCHBD-1 plasmid. C–D) HSC-3, UM1, SCC-9 and SCC-25 cells transfected by pGCHBD-1 displayed inhibited migration (C) and invasion (D). All assays were carried out three times in triplicates. E) Western blotting of tumor migration and invasion associated vimentin, RhoA, RhoC and MMP-2, GAPDH was used as an internal control. After exogenous expression of hBD-1, decreased expression of MMP-2 was observed in HSC-3, UM1 and SCC-25 cell lines, while decreased expression of RhoA and RhoC was only observed in UM1. No change of vimentin expression was observed in these cell lines. Representative blots from three independent experiments.

To verify whether hBD-1 would regulate the migration or invasion of OSCC cells *in vitro*, exogenous expression of hBD-1 was induced by lentivirus-delivered plasmid in HSC-3, UM1, SCC-9 and SCC-25. All 4 cell lines infected by pGCHBD-1 showed significantly increased hBD-1 expression compared with cells infected by pGCcontrol (Figure 3A–B). Migration and invasion assay showed that exogenous expression of hBD-1 significantly suppressed migration of all 4 cell lines (Figure 3 C) and invasion of HSC-3, UM1 and SCC-9 cell lines (Figure 3D). As shown in Figure 2F, SCC-25 cells had very low invasion ability. After infection by pGCHBD-1 or pGCcontrol, very few cells (less than 5 cells per chamber) invaded to the lower surface of the cell culture insert in either group, these data were not included in further analysis.

**Figure 4:**
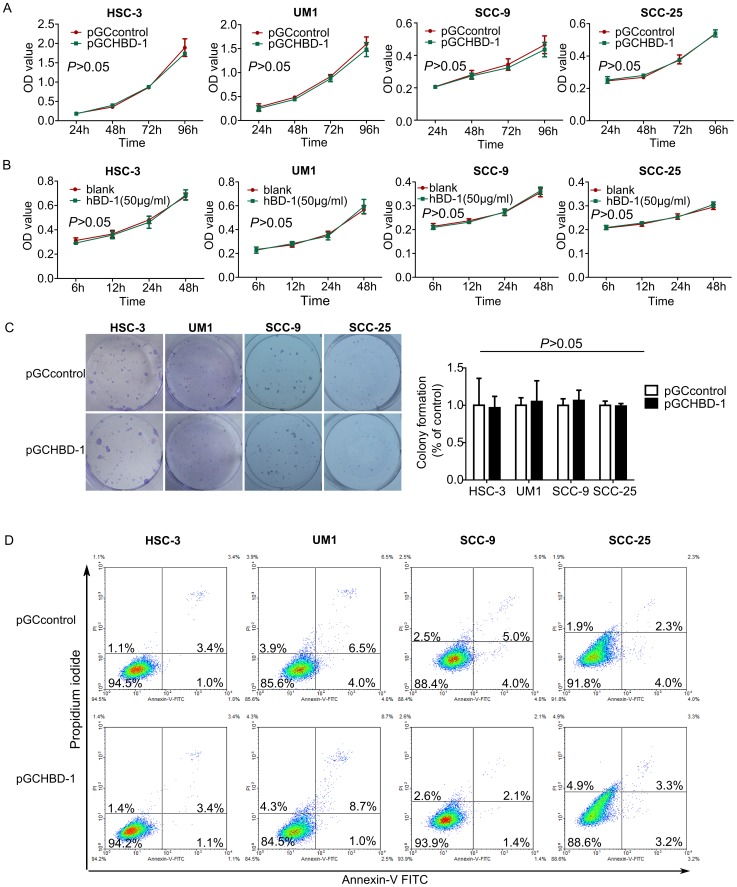
hBD-1 overexpression showed no significant effect on proliferation of HSC-3, UM1, SCC-9 and SCC-25 cells *in vitro*. A) OSCC cells transfected with pGCHBD-1 plasmid showed similar viability compared with control cells in a span of 96 hours. B) Treatment with recombinant hBD-1 protein (50 µg/ml) did not have any significant effect on the viability of HSC-3, UM1, SCC-9 or SCC-25 cells over a period of 48 hours. C) Exogenous expression of hBD-1 did not have any significant effect on colony formation ability of these two cell lines, either (left panel: crystal violet staining; right panel, relative colony number). D) Flow cytometry analysis of cell apoptosis by Annexin-V FITC and PI double staining after transfection with pGCHBD-1 or control plasmid.

To confirm whether the inhibitory effect of hBD-1 on the migration and invasion of OSCC cells was due to suppression of cell proliferation or induction of apoptosis, the effects of hBD-1 on proliferation and apoptosis of these 4 cell lines were also examined. However, neither exogenous expression of hBD-1 (Figure 4 A) nor treatment by recombinant hBD-1 protein (50 µg/ml) (Figure 4 B) had any significant effect on the proliferation of any cell line. There was no significant difference of colony formation ability between hBD-1 over-expressing cells and control cells (Figure 4 C). HBD-1 expression didn't have any significant influence on apoptosis of these cell lines, either (Figure 4 D).

To further explore the possible mechanism of the inhibitory effect of hBD-1 on migration and invasion of OSCC cells, a pilot study was carried out to determine the expression of tumor migration and invasion associated proteins after exogenous expression of hBD-1 by western blot. In the current study, vimentin, RhoA, RhoC and MMP-2 was examined (Figure 3 E). UM1 cells transfected with hBD-1 showed decreased expression of RhoA, RhoC and MMP-2 while HSC-3 and SCC-25 cells only showed decreased expression of MMP-2 compared with control cells. There was no substantial change of vimentin expression after exogenous expression of hBD-1.

### HBD-1 protein expression in OSCC tissue was an excellent predictor of cancer-specific survival of OSCC patients

**Figure 5:**
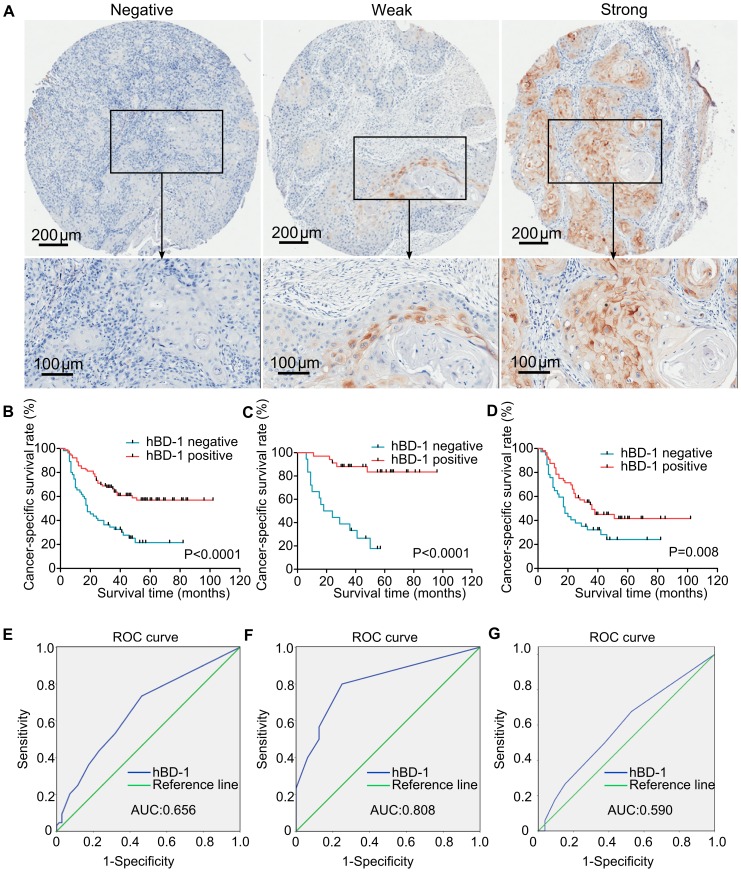
Association of hBD-1 expression with prognosis of OSCC. A) Examples of negative, weak and strong staining of hBD-1 protein in tissue core from the tissue microarray. The lower panels displayed a magnified vision of the area in the corresponding black box. B–D) Kaplan-Meier analysis displayed cancer-specific survival time of patients with OSCC, and hBD-1 expression was associated with higher survival rate in all OSCC patients (B) as well as subsets of patients with stage I–II OSCC (C) and stage III–IV OSCC (D). E–G) ROC curve analysis of hBD-1 expression as a predictor for 3-year survival of all patients with OSCC (E), patients with stage I–II OSCC (F) and patients with stage III–IV OSCC (G). An area under the ROC curve (AUC) of 0.7–0.9 is considered excellent discrimination, whereas an AUC of 0.5–0.7 is considered moderate discrimination. The ROC curve showed that hBD-1 expression had excellent discrimination for the prediction of 3-year survival in patients with early stage OSCC (stage I–II).

Finally, we investigated the prognostic value of hBD-1 expression in a tumor tissue microarray containing tumor tissues from 175 patients with primary OSCC (Figure 5 A). The mean follow-up time of these patients was 35 months (median, 33 months; range 2∼102 months). As shown in Table 1, hBD-1 expression is significantly associated with lymph node status (P = 0.043), but had no association with the tumor size (P = 0.717). Meanwhile, hBD-1 expression was also associated with differentiation status (P<0.001) and smoking history (P = 0.029), but was not associated with gender or age of the patients.

**Table 1:** Association of hBD-1 expression in OSCC with patients' clinicopathological characteristics (n = 175).

	Total n	Negative n (percentage)	Weak n (percentage)	Strong n (percentage)	*P* value
Gender					
Female	50	15 (30.0%)	24 (48.0%)	11(22.0%)	0.065
Male	125	58 (46.4%)	53 (42.4%)	14 (11.2%)	
Age (year)					
<60	85	38 (44.7%)	39 (45.9%)	8 (9.4%)	0.198
≥60	90	35 (38.9%)	38 (42.2%)	17 (18.9%)	
Smoking status					
Smoker	97	49 (50.5%)	38 (39.2%)	10 (10.3%)	0.029
Nonsmoker	78	24 (30.8%)	39 (50.0%)	15 (19.2%)	
Differentiation					
Well	107	29 (27.1%)	54 (50.5%)	24 (22.4%)	<0.001
Medium/Poor	68	44 (64.7%)	23 (33.8%)	1 (1.5%)	
pT					
T1–T2	90	35 (38.9%)	41 (45.5%)	14 (15.6%)	0.717
T3–T4	85	38 (44.7%)	36 (42.4%)	11 (12.9%)	
pN					
N0	110	39 (35.4%)	51 (46.4%)	20 (18.2%)	0.043
N1–Nx	65	34 (52.3%)	26 (40.0%)	5 (7.7%)	
Tumor stage					
I	17	5 (29.4%)	8 (47.1%)	4 (23.5%)	0.138
II	45	16 (35.5%)	21 (46.7%)	8 (17.8%)	
III	46	21 (45.6%)	20 (43.5%)	5 (10.9%)	
IV	67	31 (46.3%)	28 (41.8%)	8 (11.9%)	

The cumulative survival rate of OSCC patients with weak/strong positive staining of hBD-1 was significantly higher than those with negative hBD-1 staining (P<0.0001 by log-rank test, Figure 5 B). Cox proportional hazard model was used to assess association between cancer-specific survival of OSCC and clinicopathological factors. The hazard ratio (HR) and related 95% confidence interval (CI) of each factor was listed in Table 2. Differentiation, lymph node stage and hBD-1 expression were significantly related with prognosis in univariate analysis and were then selected in a multivariate analysis. In the multivariate analysis, the prognostic value of lymph node status (HR, 2.017; 95% CI, 1.273–3.194; P = 0.003) and hBD-1 expression (HR, 0.382; 95% CI, 0.238–0.615; P<0.001) was confirmed while the others were determined to not be prognostic factors in this cohort of OSCC patients. In order to find out whether the prognostic value of hBD-1 expression was different in different stages of OSCC, a subset analysis was done according to the UICC stages of the patients (stage I–II vs. stage III–IV). Kaplan-Meier analysis showed that OSCC patients with positive hBD-1 expression had longer survival rate in both subgroups (Figure 5 C & D, P<0.001, P = 0.008, respectively.)

**Table 2:** Cox's Proportional hazard model analysis of prognostic factors in patient with OSCC.

Variables	HR (95% CI)	Unfavorable/favorable	*P*
Univariate analysis			
Gender	0.957(0.584–1.570)	Male/Female	0.863
Age (year)	0.914(0.731–1.144)	≥60/<60	0.448
Smoking status	0.841(0.537–1.316)	Smoker/Nonsmoker	0.448
Differentiation	1.765(1.121–2.779)	Medium or Poor/Well	0.014
pT	1.482(0.944–2.325)	T3–T4/T1–T2	0.087
pN	2.040(1.302–3.196)	N1–Nx/N0	0.002
hBD-1	0.371(0.236–0.583)	Negative/Positive	<0.001
Multiviriate analysis			
Differentiation	1.161(0.716–1.882)	Medium or Poor/Well	0.546
pN	2.017(1.273–3.194)	N1–Nx/N0	0.003
hBD-1	0.382(0.238–0.615)	Negative/Positive	<0.001

To further validate the prognostic value of hBD-1 expression in OSCC, we next performed ROC curve analysis to determine the discrimination of hBD-1 expression as a predictor for 3-year survival of OSCC patients. The area under the ROC curve (AUC) was determined from the plot of sensitivity and (1-specificity) and was considered as a measure of the predictability of a test. The sensitivity of a test was defined as the true positive rate (patient would be alive after 3 years when the test was positive), while the specificity was defined as the true negative rate (patient would die after 3 years when the test was negative). AUC between 0.7 and 0.9 was considered excellent discrimination, AUC between 0.5–0.7 was considered as moderate discrimination, while AUC of 0.5 was considered as no discrimination [29]. The AUC of hBD-1 was 0.657 (Figure 5 E), which was considered as moderate discrimination. In the subset analysis according to the UICC stages, the AUC of hBD-1 expression was 0.779 for patients with stage I–II OSCC (Figure 5 F); whereas for patients with stage III–IV OSCC, the AUC of hBD-1 expression was 0.589 (Figure 5 G), indicating that hBD-1 expression had an excellent value of discrimination in patients with stage I–II OSCC but only a moderate discrimination in stage III–IV OSCC.

## Discussion

HBD-1 is an antimicrobial peptide that is mainly expressed in epithelial tissues. Recently, it has been considered as a potential tumor suppressor in renal cell carcinoma and prostate cancer [17], [18].

In the current study, it was the first time that expression of hBD-1 protein had been analyzed in the oral mucosa at different stages of oral carcinogenesis. Unexpectedly, we did not find a continuous decreasing pattern of hBD-1 expression from normal oral mucosa to OSCC, as displayed in other cancer types [15]–[18]. Instead, there wasn't any detectable expression of hBD-1 protein in all the 15 samples of normal oral mucosa, indicating that hBD-1 was not expressed in normal oral mucosa, or at least was expressed at such a low level that could not be detected by immunohistochemistry. These results were quite different from several previous studies, in which hBD-1 was reported to be constitutively expressed in oral mucosa [7], [19], [21]. However, in most of these studies, only the expression of hBD-1 mRNA was examined, which could not precisely reflect the expression level of hBD-1 protein. Though the expression of hBD-1 in normal oral mucosa was not detectable, it was observed in some of the OLK and OSCC samples, with a decreasing proportion of positive hBD-1 staining from OLK to OSCC. The reason why hBD-1 expression rate increased in precancerous lesions like OLK but then decreased in OSCC remained unclear to us. A previous study by Joly et al. showed that, although hBD-1 expression in oral keratinocytes could not be induced by pro-inflammatory cytokines such as interleukin-1β (IL-1β), IL-2, IL-6, IL-8, IL-12 and tumor necrosis factor-α, it could be induced by interferon-γ (IFN-γ) [30]. Other studies using different cell lines also demonstrated that hBD-1 expression could be increased by stimulation of IFN-γ or lipopolysaccharide [31], [32]. Therefore, a possible speculation is that expression of hBD-1 in OLK and OSCC may be a response of the body to inflammatory stimulus. Further study is warranted to prove this hypothesis.

Moreover, the proportion of hBD-1 positive samples in OSCC with metastasis was lower than those without metastasis; hBD-1 expression in OSCC cell lines differed from each other and was negatively associated with the invasive potential of the OSCC cells. Taken together, these findings raised the suspect that hBD-1 might have some regulatory effect on the migration and invasion of OSCC cells. In fact, exogenous expression of hBD-1 by lentivirus-delivered plasmid led to inhibition of migration and invasion of four OSCC cell lines *in vitro*. The inhibitory effect of hBD-1 on cancer cells, as well as the induction of apoptosis by hBD-1 in a caspase- related way had been reported by Sun [17] and Bullard [18], [33]. However, in the current study, we hadn't observed any significant effect of hBD-1 on the proliferation or apoptosis of OSCC cell lines HSC, UM1, SCC-9 and SCC-25. This is in accord with a previous report, in which Nishimura and colleagues [34] showed that hBD-1 had no effect on the proliferation of OSCC cell line KB (which is actually a derivative of Hela cells [35]) and SCC-9. Although in another report a mild inhibitory effect of hBD-1 on OSCC cell line BHY was observed [24], we could at least inferred from our results that hBD-1 did not have universal inhibitory effect on the proliferation of OSCC cells. These findings were then supported by the clinicopathological data, in which hBD-1 expression was significantly related with lymph node status but not tumor size.

The precise mechanism of how hBD-1 regulates the migration and invasion of OSCC cells is still un-known. In our pilot study, we detected the expression of 4 proteins which were closely related with the invasive potential of tumor cells, including vimentin, RhoA, RhoC and MMP-2 [36]–[40]. Decreased expression of MMP-2 was observed in HSC-3, UM1 and SCC-25 cell lines after exogenous expression of hBD-1, while decreased expression of RhoA and RhoC was only observed in UM1 cells. There wasn't any significant change of the expression of vimentin. These findings suggest that MMP-2, probably RhoA and RhoC may be associated with the regulatory effect of hBD-1 on the migration and invasion of OSCC cells, but further validation is needed.

Another main finding of this study is the significant correlation of hBD-1 expression with cancer-specific survival of OSCC patients. This is the first time that hBD-1 has been reported as a predictor for the prognosis of OSCC, or any type of cancer. Clinicopathological evidence from our TMA indicated that OSCC patients with hBD-1-positive tumors had higher survival rate and longer cancer-specific survival time. Multivariate analysis confirmed hBD-1 expression as an independent prognostic factor of cancer-specific survival besides lymph node status for patients with stage I–II OSCC. The prognostic value of hBD-1 as a predictor for OSCC survival was further validated by ROC curve analysis, in which area under the ROC curve was used as a measure of the predictability of the predictor [29]. Although hBD-1 expression showed only moderate discrimination for the prediction of 3-year survival of OSCC patients as a whole, it did show excellent discrimination in patients with stage I–II OSCC. Taken together, these data suggested that hBD-1 expression was associated with cancer-specific survival of OSCC, and it was a potential predictor for the survival of patients with primary OSCC, especially OSCC at early stage.

In summary, we reported about a decreasing pattern of hBD-1 expression from oral precancerous lesions to OSCC, a relationship between hBD-1 expression and invasive potential of OSCC and an inhibitory effect of exogenous expression of hBD-1 on the migration and invasion of OSCC cells. Moreover, our study is the first to show that hBD-1 expression is associated with cancer-specific of OSCC and can be a potential predictor of survival of early stage OSCC. These results suggested that hBD-1 might inhibit the progression of OSCC by suppressing the migration and invasion of OSCC cells and could be further investigated as a potential therapeutic target as well as a prognostic marker in patients with OSCC.

## Supporting Information

Figure S1 **Dot blot indicating specificity of anti-hBD-1 antibody.** 200 ng of synthetic hBD-1 and hBD-2 were blotted onto the polyvinylidene difluoride (PVDF) membrane (Millipore). Following protein transfer, the membrane was incubated in 5% bovine serum albumin containing anti-hBD-1 antibody (mouse monoclonal, Abcam) followed by horseradish peroxidase conjugated goat anti-mouse antibody and visualized with an ECL detection system (Pierce). The result indicates positive reaction of this antibody against synthetic hBD-1 peptide without cross reaction with hBD-2 peptide.
(TIF)
