# Supplementary figures and images for: Human Beta-Defensin-1 Suppresses Tumor Migration and Invasion and Is an Independent Predictor for Survival of Oral Squamous Cell Carcinoma Patients
Source: PLoS One. 2014 Mar 21;9(3):e91867. doi: 10.1371/journal.pone.0091867 (PMC3962354; doi:10.1371/journal.pone.0091867)

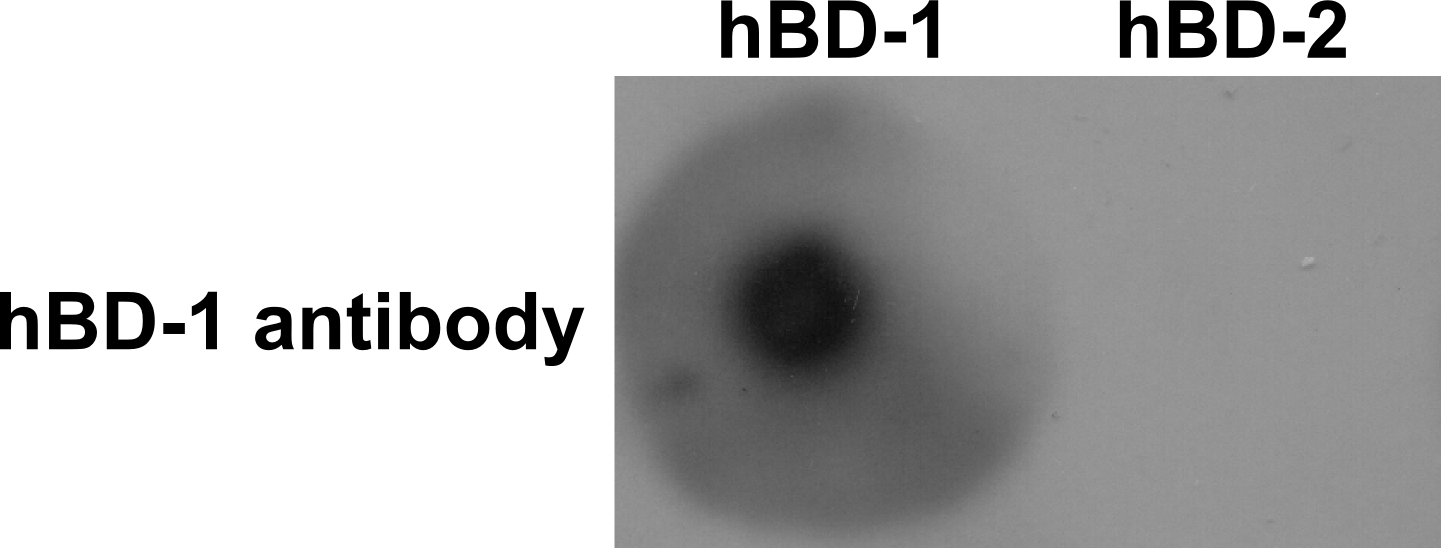

Supplement: Figure S1 — Dot blot indicating specificity of anti-hBD-1 antibody. 200 ng of synthetic hBD-1 and hBD-2 were blotted onto the polyvinylidene difluoride (PVDF) membrane (Millipore). Following protein transfer, the membrane was incubated in 5% bovine serum albumin containing anti-hBD-1 antibody (mouse monoclonal, Abcam) followed by horseradish peroxidase conjugated goat anti-mouse antibody and visualized with an ECL detection system (Pierce). The result indicates positive reaction of this antibody against synthetic hBD-1 peptide without cross reaction with hBD-2 peptide. (TIF) [file pone.0091867.s001.tif]
